# Janus-Faced Molecules against Plant Pathogenic Fungi

**DOI:** 10.3390/ijms222212323

**Published:** 2021-11-15

**Authors:** Gaspar Banfalvi

**Affiliations:** Department of Molecular Biotechnology and Microbiology, Faculty of Science and Technology, University of Debrecen, 1 Egyetem Square, 4010 Debrecen, Hungary; bgaspar@unideb.hu or gaspar.banfalvi@gmail.com; Tel.: +36-52-512-900 (ext. 62319) or +36-20-5818325

**Keywords:** mycotoxins, classification of carcinogens, animal models, agents against plant mycoses, structure–function relationship

## Abstract

The high cytotoxicity of the secondary metabolites of mycotoxins is capable of killing microbes and tumour cells alike, similarly to the genotoxic effect characteristic of Janus-faced molecules. The “double-edged sword” effect of several cytotoxins is known, and these agents have, therefore, been utilized only reluctantly against fungal infections. In this review, consideration was given to (a) toxins that could be used against plant and human pathogens, (b) animal models that measure the effect of antifungal agents, (c) known antifungal agents that have been described and efficiently prevent the growth of fungal cells, and (d) the chemical interactions that are characteristic of antifungal agents. The utilization of apoptotic effects against tumour growth by agents that, at the same time, induce mutations may raise ethical issues. Nevertheless, it deserves consideration despite the mutagenic impact of Janus-faced molecules for those patients who suffer from plant pathogenic fungal infections and are older than their fertility age, in the same way that the short-term cytotoxicity of cancer treatment is favoured over the long-term mutagenic effect.

## 1. Introduction

The Roman god Janus is depicted as having two faces, one facing the past and the other looking into the future [1]. Double-edged swords, also referred to as Janus-faced molecules, represent both beneficial and toxic effects and are widespread in biology; however, their molecular mechanisms are only poorly clarified. Among the double-edged swords are curcumin, known for its cancer-preventive and therapeutic role [2], nitric oxide [3], cholesterol [4], and peptide nucleic acid (PNA) [5]. Mycotoxins are regarded as “Janus-faced” molecules due to their adverse effects. The definition of Janus-faced molecules refers to the harmful genotoxic (mutagenic, teratogenic, and carcinogenic) effect [6,7] as well as the cytocidal and antineoplastic effects [8,9,10].

The prevalence of fungal infections is increasing worldwide due to the increase in the ageing population and patients with compromised immune systems. Cases of opportunistic mycoses are known to increase each year [11]. The ubiquitous plant and human pathogenic conditions aspergillosis, fusariosis, and cryptococcosis were known earlier as exceptionally rare human diseases. The frequency of aspergillosis has especially increased in patients without weakened immune systems but who have severe respiratory infections caused by viruses, such as influenza. Recent reports describe COVID-19-associated pulmonary aspergillosis [12,13,14,15,16,17,18,19]. *Fusarium* secondary metabolites are sporadically present in livestock feeds and may cause a variety of disorders termed “mycotoxicoses”, some of which are fatal [20]. Mycotoxins are carcinogenic toxins that are produced by many *Aspergillus* and *Penicillium* species growing on food commodities [21]. In the past, it seemed unrealistic to treat saprophytes and plant pathogenic fungi with expensive antifungal agents, such as antibiotics. This attitude changed in the last decades since it was discovered that plant pathogenic fungi may cause fatal infections. The most frequently occurring lethal infection is aspergillosis, especially in neutropenic patients with hematologic malignancy [22]. Disseminated fusariosis is the second most frequent lethal fungal infection [23,24,25].

Experiments related to the antifungal activity of antibiotics against plant pathogenic fungi were carried out in the 1970s but were not practical and remained unpublished until the retrospective evaluation of gentamicins against *Fusarium* species [24]. Major fractions of the gentamicin complex (C1, C1a, C2, C2a) are toxic and possess only moderate antifungal activity. However, among minor gentamicin components (A, A1–A4, B, B1, X), gentamicin B1 was found to be an efficient antifungal agent in vitro, maintaining some of its useful in vivo antifungal potential in mice, either alone or in combination with amphotericin B [25].

Gentamicin B1 is a minor gentamicin component that was reported to be a major nonsense mutation suppressor [25,26]. In addition to gentamicin, the G418 readthrough inducer was reported to counteract the effects of nonsense mutations [26,27]. However, it turned out that the synthetic aminoglycoside G418, closely related to gentamicin B1, was not an inducer of premature termination codon readthrough [26]. Gentamicin B1 and G418 (geneticin) differ only in the location of C2 of the purpuroseamine ring by the substitution of a hydroxyl group in gentamicin B1 by an amino group in G418. The gentamicin B1 compound used in this study was acquired commercially and was not gentamicin B1 but the closely related aminoglycoside G418 [28]. We used gentamicin fractions, among them gentamicin B1, that were separated, purified, and characterized, and their structures were determined by established protocols [29,30].

Toxins produced by plant and human pathogenic fungi, as well as agents with functional groups exerting demonstrated antifungal effects on these species, are overviewed in this paper. More attention is given to the toxins of the more aggressive *Aspergillus* species than to other plant pathogenic fungi, such as *Fusarium* and *Cryptococcus*. In immunocompetent individuals, the conidia of *Aspergillus fumigatus* are effectively eliminated by the innate immune system, but in immunosuppressed patients, they cause mainly serious pulmonary diseases [31]. The presence of these toxins in food and feed diminish liver and/or kidney functions. *Aspergillus*-derived mycotoxins in the feed and food chain have been recently reviewed in several publications [32,33,34,35,36,37,38,39,40,41,42,43,44,45,46,47,48,49,50,51].

### 1.1. Aspergillus Toxins

**Figure 1:**
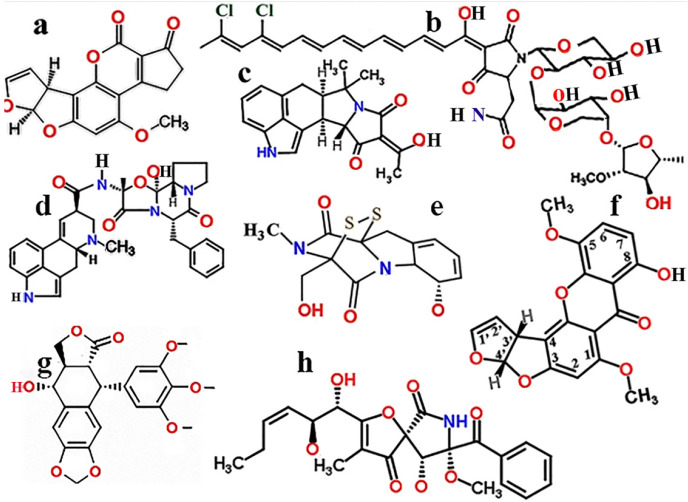
Major carcinogenic *Aspergillus* mycotoxins. Polyaromatic: aflatoxin (**a**), aliphatic compounds: aurantosides (**b**), cyclopiezonic acid (**c**), ergotamine (**d**), podophyllotoxin (**e**), gliotoxin (**f**), stigmatocystins (**g**), pseurotin (**h**). Modified with permission [32].

Mycotoxigenic fungi [52,53] belong mainly to three genera, the *Aspergillus*, *Fusarium*, and *Penicillium* species [53,54]. The most dangerous mycotoxins are carcinogenic compounds (Figure 1).

The International Agency for Research on Cancer (IARC) (Lyon, France) categorized human carcinogenic mycotoxins and found that only aflatoxins belong to the most carcinogenic IARC 1 category (Figure 1a). The dicumarin derivative aflatoxins produced mainly by *Aspergillus flavus* and *A. parasiticus* were isolated as B1, B2, G1, and G2 aflatoxins. The B and G aflatoxins exhibit blue and green fluorescence under UV light [55]. It is self-evident that polyaromatic carcinogenic aflatoxins cannot be used against cancer. Other less toxic mycotoxins containing simple aromatic rings are less toxic and often found in physiological compounds, such as five-membered heterocyclic rings, like furane or pyrrole, or six-membered rings, such as benzene or heteropolycyclic pyridine. Fused/condensed aromatic rings, consisting of monocyclic rings and sharing their connecting bonds, deserve even more attention as potential cytotoxic agents.

### 1.2. Structural and Toxic Similarities of Aspergillus Mycotoxins

The functional and structural similarities of *A. fumigatus* mycotoxins are illustrated in Figure 1. The carcinogenic properties of mycotoxins are exemplified by polyaromatic aflatoxins and aliphatic aurantosides, as shown in Figure 1ab [32,56,57]. *Aspergillus* toxins, such as ergot alkaloids, especially from *A. Fumigatus,* cover a wide range of biological activities with effects on blood circulation and neurotransmission, to mention the most important ones [58,59]. The permeable ergot-like alkaloid cyclopiazonic acid isolated from *Aspergillus flavus* and *Aspergillus versicolor* exerts reversible inhibition on the sarcoendoplasmic (SERCA) reticulum and sarcoplasmic Ca^2+^ ATPases [60]. 

The secondary metabolite pseurotin of *Aspergillus* and other fungi [60,61] are induced in response to hypoxia [61,62]. Pseurotins are characterized by an unusual spirocyclic furanone–lactam core and have different biological activities, including the modulation of the immune response. Pseurotin A showed a moderate effect against phytopathogenic bacteria and presented low cytotoxicity toward human lung fibroblasts [63]. Pseurotin and its analogue, synerazol, are inhibitors of immunoglobulin E [64]. Natural pseurotins inhibit not only the activation of B-cells into plasma cells [65] but also the activation of lymphocyte proliferation [66] and inflammatory responses, inactivating the STAT signalling pathways in macrophages [67]. More importantly, the anticancer agent pseurotin A became known as a novel suppressor of hormone-dependent breast cancer progression [68]. Pseurotin A isolated from *Aspergillus fumigatus* also possesses antioxidant properties and was recently shown to exhibit a wide range of potential therapeutic applications [69].

Among the xanthene-derivative sterigmatocystins, the mycotoxin 5-5-MS was isolated from an *Aspergillus* sp. strain, and it was found that the active substance 5-MS possessed a strong antineoplastic effect [70]. It is associated with the bisfurano ring and the double bond in the highly cytotoxic mycotoxins, has tolerable side effects, and could potentially act against metastasis [32]. The ‘Janus-faced’ terminal furan ring, which is also present in aflatoxins, is produced mainly by *Aspergillus flavus* and *Aspergillus parasiticus*, but the carcinogenic side effect of aflatoxins makes them unsuitable as antimetastatic agents.

**Figure 2:**
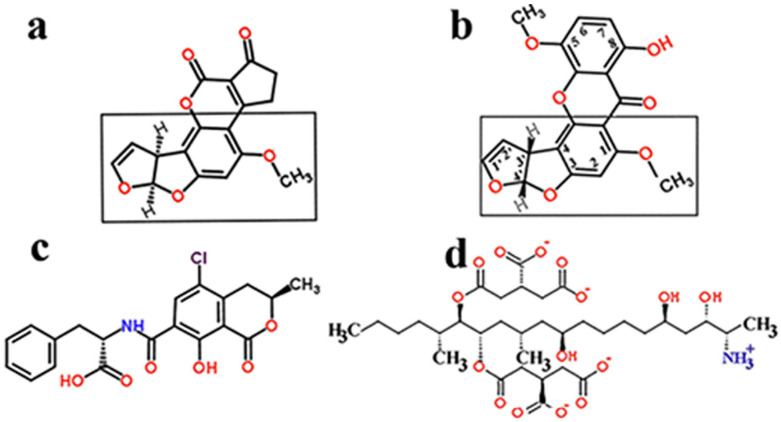
Structural similarities and IARC carcinogenic classification of major *Fusarium* mycotoxins. (**a**) aflatoxin B1—IARC I category, (**b**) 5-methoxysterigmatocystin (5-MS)—IARC 2B category, (**c**) ochratoxin A—IARC 2B, (**d**) fumonisin B1—IARC 2B. Identical structural parts of aflatoxin B1 and 5-MS are boxed.

The toxin of the marine fungus *Aspergillus terreus* induces changes in metabolism [70,71]. The structural relationship suggests that, after aflatoxin B1 (Figure 2a), 5-MS (Figure 2b) appears to be the second most dangerous mycotoxin, possibly belonging to the carcinogenic IARC 2B category.

The cytotoxicity of mycotoxins is definitely attributable to their multiple hydroxyl groups, whereas the carcinogenic properties are attributable to their polyene structure and cumulative presence of keto groups. The reduction in toxicity is discussed in the section on basic water-soluble antibiotics. 

The structure of IARC carcinogens, such as ochratoxins and fumonisins, confirms that mutagenicity is contributed primarily by the keto, hydroxy, and the cumulative presence of carboxy groups (Figure 2c,d). Mycotoxins belonging to higher IARC numbers are not classifiable as to their carcinogenicity to humans. Major mycotoxins belonging to the non-carcinogenic IARC 3 category are nivalenol, deoxynivalenol, patulin, T-2/HT-2 toxins, zearalenone [22]. T-2 and HT-2 are the toxins produced primarily by *Fusarium* sporotrichioides, which occur in small grains. Patulin is a poison produced by the *Aspergillus* and *Penicillium* species. Patulin is present in apples, its amount correlating with the degree of rot in apples.

Besides the structural similarities of carcinogenic *Aspergillus* toxins, the polyene ring structures of aromatic compounds, as well as the polyhydroxy and polycarboxylic substituent aliphatic fumonisin B1, deserve mention. Ochratoxin A is nephrotoxic and hepatotoxic to animals and humans and is a potent teratogen, carcinogenic, and immune suppressant [72,73,74].

### 1.3. Fusarium Toxins

**Figure 3:**
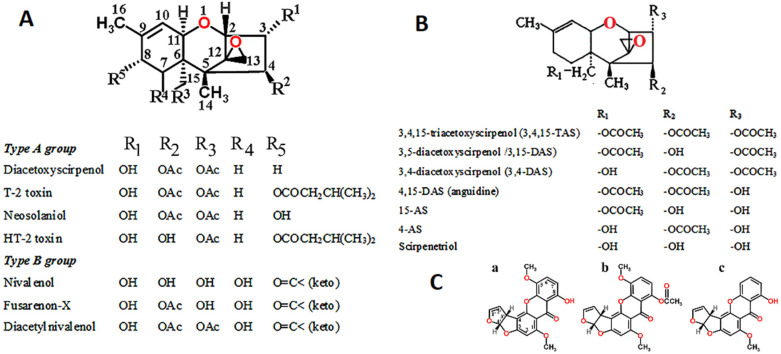
*Fusarium* toxins. Type A trichothecenes: (**A**) diacetoxyscirpenol, T-2 toxin, neosoleniol, HT-2 toxin. Type B trichothecene group: nivalenol, fusarenon-X, diacetylnivalenol. (**B**) Anguidine analogues of trichothecenes: 3,4,15-TAS; 3,-15 DAS; 3,4-DAS; 15 DAS; 4,15-DAS (anguidine); 15-AS; 4-AS; scirpenetriol. (**C**) The chemical structure of antileukemic 5-methoxysterigmatocystin (5-MS) derivatives. (**a**) 5-MS, (**b**) O-acetyl-5-MS, (**c**) sterigmatocystin. The ring structure consists of 2 five-membered bisfurano rings and 3 six-membered rings forming the xanthone skeleton. Metabolic pathways of *Fusarium* mycotoxins are diversified. Formulas permitted by [32].

Mycotoxin-producing *Fusarium* species are *F. sporotrichioides*, *F. graminearum*, and *F. verticillioides*. The structures of major toxins include zearalenone, zearalene, deoxynivalenol, nivalenol, T-2 toxin, and diacetoxyscirpenol (Figure 3) [75].

Trichothecenes A, B, C, and D are synthesized by several fungal genera, but only types A and B are produced by *Fusarium* spp. Types C and D trichothecenes have different chemical structures and are not produced by Fusarium species [76]. The Fusarium-produced trichothecenes include simple as well as complex mycotoxins, containing a single six-membered ring in the type A and type B trichothecenes, the anguidine analogues, and the antileukemic 5-methoxysterigmatocystin (5-MS) derivatives (Figure 3 [77,78]). All trichothecenes containing the core six-membered ring are flanked by a single oxygen atom in an epoxy constellation [66], which makes it strained and highly reactive. The major type B trichothecenes, including deoxynivalenol, nivalenol, and acetyldeoxynivalenol mycotoxins, induce pathological lesions, primarily necrosis of the intestinal epithelium [79]. The problem with the distribution is the identification of Fusarium isolates at the species level. Further improvement of the mass spectrometry database is needed to enable the precise identification at the species level of any Fusarium isolates encountered either in human pathology or in the environment [80].

As far as anguidine analogues are concerned (Figure 3B), the in vivo antitumor activity in mice was measured upon treatment by triacetoxyscirpenol, the three diacetoxyscirpenols, the three monoacetoxyscirpenols, and scirpenetriol [81].

The 5-methoxysterigmatocystin (Figure 3C) is produced by *Aspergillus versicolor* and *A. flavus* and is known for its antineoplastic property, with high cytotoxicity and genotoxicity characteristic of Janus-faced molecules. Although 5-methoxysterigmatocystin was investigated [82], it was not suitable for diagnostic or therapeutic use due to its hepatotoxicity and carcinogenic properties (https://www.scbt.com/p/5-methoxysterigmatocystin-22897-08-1 (accessed on 05 November 2021) (SCBT—Santa Cruz Biotechnology-scbt.com, Dallas, TX, USA).

### 1.4. Cryptococcus Toxins

Cryptococcosis is a potentially fatal fungal disease caused by the toxins of a few species of *Cryptococcus,* primarily *C. neoformans* and *C. gattii. C. gattii*, unlike *C. neoformans,* prefers to grow in a moist environment. *Cryptococcus neoformans* is the major pathogenic species in both human and animal hosts. *C. neoformans* secretes a large variety of enzymes with the potential to degrade host molecules [83].

Among the enzymes produced by the fungus, the major candidates as mediators of host toxicity at the molecular level are proteases, ureases, phospholipases, and nucleases. *C. neoformans* can metabolize immunoglobulins and complement proteins for growth, as these compounds are presumably degraded by the released proteases [84].

### 1.5. Toxins Inhibiting the Growth of Aspergillus Species

Aurantosides are cytotoxic tetrameric acid glycosides isolated from marine *Theonella* species and exhibit the most efficient antifungal activity against *Aspergillus fumigatus* besides the yeast *C. albicans* [85,86].

## 2. Animal Models to Test Mycoses Caused by Plant Pathogenic Fungi

Among the reproducible rodent models of invasive aspergillosis, the murine model is the most popular and frequently used, followed by the rat models These models are suitable for testing fungal virulence, infection pathogenesis, diagnostic markers, and testing the antifungal therapy of invasive aspergillosis with relative ease of manipulation, economy, and handling of a large number of reagents. Murine models remain the most commonly used models for studying aspergillosis due to the ease of manipulation and the large number of reagents available for studying disease-host responses [87].

Invasive fungal infections are the major cause of death in organ transplant patients. Invasive human aspergillosis was mimicked by immunosuppressing mice with cyclophosphamide and cortisone acetate and then infecting them in an aerosol chamber. This procedure reproducibly delivered 10^3^ to 3 × 10^3^ conidia to the lungs. Lethal pulmonary aspergillosis developed in 2 weeks but could be prevented by amphotericin B. The murine hydrocortisone-mediated immunosuppression model of pulmonary aspergillosis is commonly used to characterise invasive fungal infections. However, this model does not take into account the effects of the fungal calcineurin pathway, which is required for both virulence and antifungal drug resistance. A new and clinically relevant transplant immunosuppression model showed that patients with pulmonary aspergillosis were immunosuppressed with calcineurin inhibitors and steroids [88]. Due to the growing importance of plant pathogenic fungi and their human diseases causing high morbidity in humans and animals, aspergillosis, over recent decades, has created a need for practical and reproducible models.

Airborne fungal disease, generated by plant pathogenic ubiquitous moulds, causes high morbidity and mortality in both human and animal populations. The growing significance of these infections in the last 50 years is demonstrated by the interest in efficient in vitro treatment of plant pathogenic fungi. Recent experiments relate to reproducible experimental rodent models of systemic candidiasis, meningeal cryptococcosis, pulmonary aspergillosis, and disseminated fusariosis.

These include improved murine models adapted to combat pulmonary aspergillosis [89]. The first murine model of aspergillosis was published in 1967 [89]. Early models [90,91] included intranasal [91], intratracheal administration [92], and inhalation of *Aspergillus* conidia [89,90,91,92,93,94,95,96].

**Figure 4:**
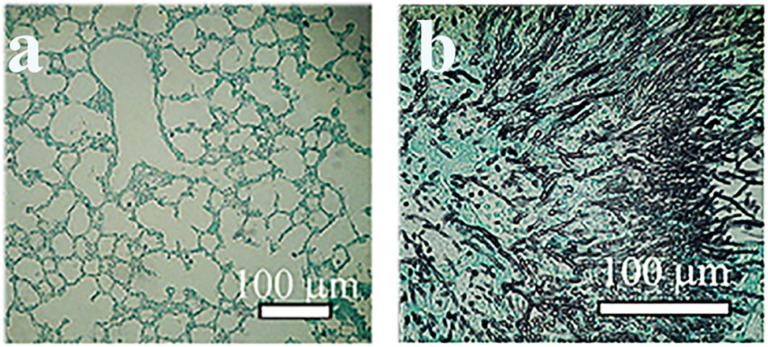
Grocott’s methenamine silver (GMS) staining to detect aspergillosis in the lung tissue of mice. (**a**) Control lung section. (**b**) Staining of lung section in immunocompromised mice 96 h after infection. Modified with permission [94].

Rodent models are intended to provide a more efficient diagnosis, involve intravenous treatment, and improve the quality of images of histological examinations by image analysis [97]. Standardization included the lossless compression to JPEG format images, using a NIKON D5100 camera, with a 1/25-s exposure, the ImageJ open-source software package, fast Fourier transformation, and bandpass filtering to avoid uneven illumination artefacts (bulb-effect removal). Local contrast was enhanced by using the histogram equalization method [98] and versatile wand tool for specific GMS staining (https://imagej.nih.gov/ij/plugins/versatile-wand-tool/index.html (accessed on 05 November 2021). As an example, Grocott’s methenamine silver staining of murine aspergillosis after image analysis is presented in Figure 4.

### Detection of Serum Beta (1-3)-D-Glucan

The fungal cell wall constituent, triple helical lentinan, a linear beta (1-3)-D-glucan, has received FDA approval, is highly sensitive, and serves as a specific test for invasive deep mycosis. The presence of this glucan can be traced in the cell wall in beta (1→3)-D-glucanaemia in deep mycosis [99]. This test could be useful in immunocompromised patients, including those infected with plant pathogenic fungi, such as fusariosis or aspergillosis. The visual nature of the plant–fungal monitoring makes digital image processing techniques adaptable to solve this problem. [100]. Adherence and growth patterns of *Candida albicans* yeast cells strains were performed earlier [101]. Recently, time-lapse image analysis has been extended to plant pathogenic fungi.

## 3. Treatment of Plant Pathogenic Fungal Infections

Non-invasive bronchopulmonary diseases may lead to invasive pulmonary aspergillosis even in immunocompetent individuals [102]. The incidence of aspergillosis rose due to infections with A. fumigatus and other pathogenic *Aspergillus* species [103]. The most frequently occurring pathogenic fungal infections (aspergillosis, fusariosis, and cryptococcosis) are difficult to treat because patients are immunosuppressed and resistant to antifungal agents [24,104].

The pulmonary aspects of cryptococcosis are often overlooked because the manifestation of cryptococcal infection is meningoencephalitis, although the initial pathogenetic event is pulmonary infection. *Cryptococcus gatti* and *C. neoformans* are the etiologic agents that cause major systemic pulmonary infections [105].

Although the mechanism of infection by fungal spores in mammalian hosts remains largely unknown, the infections of immunocompromised patients of disseminated fusariosis in immunosuppressed mice resemble patients infected by *Aspergillus* spores. Symptoms of immunosuppressed mice are also remarkably similar to those reported in humans. *Fusarium* species, such as F. oxysporum, can disseminate and persist in the organs of immunocompetent animals, and these latent infections can lead to lethal systemic fusariosis if the host is later subjected to immunosuppressive treatment [106,107,108,109,110,111,112,113,114,115].

### 3.1. Therapy of Invasive Plant Mycoses

#### Aspergillosis and Cryptococcosis

*Fusarium* spp. is an opportunistic pathogen that causes potentially fatal infection in immunocompromised patients. The mean minimal inhibitory concentration (MIC) against Fusarium of applied amphotericin B was 3.3 µg/mL, and of miconazole, 5.3 µg/mL. Patients recovered after receiving amphotericin B plus 5-fluorocytosine [116]. Posaconazole was efficient in the treatment and prophylaxis against *Fusarium solani* [117]. Pentamidine is active in a neutropenic murine model of acute invasive pulmonary fusariosis [106]. 

The following therapeutic guidelines apply:

**-**  Infection with conidial spores of *Aspergillus* species should be avoided.
**-**  Filtered air should be introduced into the air-conditioning systems in bone and marrow transplant units.
**-**  Invasive aspergillosis can be effectively treated with azoles, especially with voriconazole.
**-**  Due to the relatively fast-developing resistance, the combined therapy of voriconazole with amphotericin B or in combination with other fungal agents is recommended.
**-**  Double and triple antifungal combinations against clinical isolates of *Aspergillus fumigatus* and *A. terreus* are recommended. Combinations of caspofungin with either amphotericin B or voriconazole were additive for all the isolates, and antagonism was not observed. In contrast, the interaction between voriconazole and fluorocytosine was not synergistic; rather, antagonism was noted for 93% of the isolates [116].
**-**  Prophylactic measures should be taken to prevent the infection of the personnel.

## 4. Functional Groups of Aminoglycosides against Plant Pathogenic Fungi

**Figure 5:**
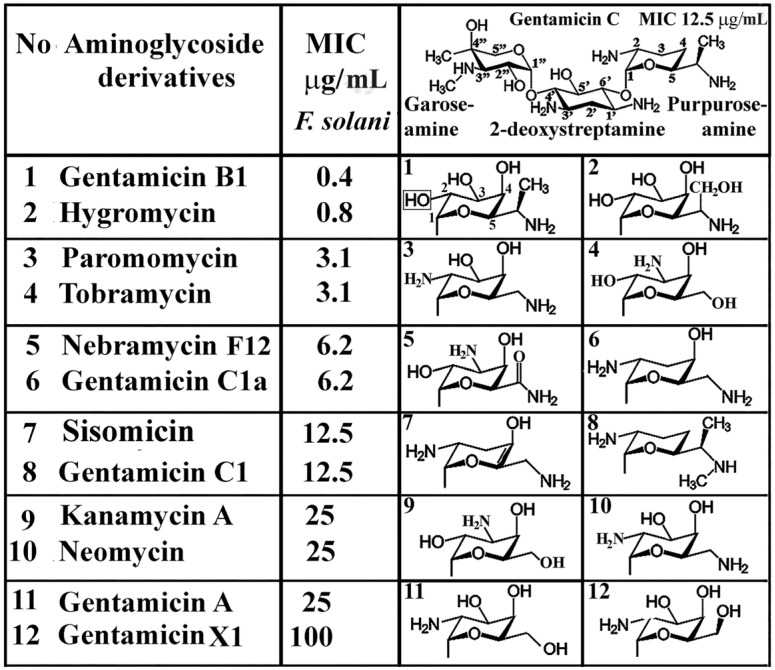
Antifungal activity of aminoglycosides on Fusarium solani. The ring structure of gentamicin C consists of garoseamine, 2-deoxystreptamine, and purpuroseamine. The substituents of purpuroseamine derivatives are given for aminoglycosides that are numbered (1–12). In the figure, only the structure of the aminoglycosides that consist of 3 rings are given. Modified with permission of [26].

Gentamicin is a broad-spectrum aminoglycoside antibiotic produced by *Micromonospora purpurea* bacteria and is effective against Gram-negative bacterial infections. Major fractions of the gentamicin complex (C1, C1a, C2, C2a) possess weak antifungal activity, but one of the minor components (A, A1-A4, B, B1, X) of gentamicin B1was found to exert strong in vitro antifungal activity against plant pathogenic fungi [25]. The in vitro antifungal activity of aminoglycosides, among them gentamicin major and minor fractions, was detected with gentamycin B1 as the most active minor fraction. The effect of basic water-soluble antibiotics, including gentamicin fractions, has been recently described [24,25]. The structure and function relationship of aminoglycosides, including the fractions of gentamycin, are summarised in Figure 5.

The in vitro antifungal potency of gentamicin B1 turned out to be higher than other established antifungal agents, including amphotericin B, clotrimazole, nystatin, and griseofulvin [24].

This relationship is placed in a broader perspective, as it suggests that not only the kinship of aminoglycoside ring structures and their substituents need consideration but the structures of other mycotoxins do as well. As a general tendency, the toxicity of mycotoxins depends largely on the oxidation state, with -OH groups responsible for the toxicity, and keto groups for the apoptotic effect. In aminoglycosides, ring structures are not aromatic and do not contribute to genotoxicity, but polyhydroxy and carbonyl (=CO) groups do contribute. It is worth mentioning that aminoglycosides, including gentamicins, are much less toxic than one would expect from oxygen-containing substituents. The purpuroseamine ring substituents of gentamicins (Figure 5) in those aminoglycoside derivatives (hygromycin, paromomycin, and neomycin) consist of four rings. The presence of the fourth ring and its substituents does not significantly impact the antifungal effect. The antifusarial spectrum of aminoglycosides [26] resolved several doubts concerning the structure–function relationship of gentamicins. The critical element of the antifusarium effect takes place at the purpuroseamine ring of the gentamicin complex and not at the garoseamine and 2-deoxystreptamine units (upper right panel of Figure 5). The substituents of the garoseamine and 2-deoxystreptamine rings are identical in each gentamicin, but their MIC values are different, suggesting no involvement of these rings in function. 

It was shown previously that the antifungal activity of gentamicin B1 on *Fusarium* species affects other plant pathogen fungi, such as *Aspergillus, Fusarium, Cryptococcus, Microsporum,* and *Trichophyton* culture, much more so than the yeast *Candida albicans* [24]. The antifungal activities of different agents besides gentamicin B1, including clotrimazole, amphotericin B, nystatin, and griseofulvin [25], showed that some of the MIC values of gentamicin B1 for *Trychophyton* and *Candida albicans* were higher than those of amphotericin B and clotrimazole. Gentamicin B1 was more efficient against *Fusarium*, *Aspergillus, Microsporum,* and *Cryptococcus* species than other established antifungal agents, such as clotrimazole, amphotericin B, nystatin, or griseofulvin. This could mean that the variability of the antifungal effect depends not only on the chemical composition of gentamicin but also on the types of plant fungi tested, and it varies with different batches of mycotoxins. The soil dermatophyte *Microsporum gypseum* is often difficult to distinguish from *Trichophyton gypseum.* Based on its inhibition pattern, *Microsporum gypseum* is more susceptible (MIC 3.1 µg/mL) to gentamicin B1 than *Trichophyton gypseum* (MIC 25 µg/mL) [25]. Based on this observation, gentamicin B1 could serve as a differential diagnostic tool to distinguish between *Trichophyton gypseum* and *Microsporum gypseum*. 

Murine experiments proved that gentamicin B1 maintained some of its antifungal effects against experimental cryptococcal infection. A combined gentamicin B1 and amphotericin B treatment, as well as their repeated application, could reduce the toxicity of elevated concentrations of gentamicin and improve the survival of patients [25]. Gentamicin as a marketed medicine might be tested against cryptococcosis and systemic plant mycoses that are still lacking efficient medical treatment. 

## 5. Discussion

Three types of antifungal agents have been reviewed in this study, including ’Janus-faced’ mycotoxins, aminoglycoside molecules as double-edged swords, and the efficient minor fraction gentamicin B1. Particular attention was given to their antifungal activity, and they were compared with the classical antifungal drugs (amphotericin B, clotrimazole, nystatin, and griseofulvin). Mycotoxins are poisonous, ubiquitous in nature, and produced by various fungi species whose occurrence in the food chain is inevitable. Therefore, they pose a serious problem on a global scale [117,118]. Human exposure to mycotoxins is commonly due to food and feed contamination [119,120]. The double-edged sword effect of antineoplastic mycotoxins has been reviewed with respect to selected mycotoxins, which affect eukaryotic cells with a broad range of structural and functional groups, contributing to their classification. The strong toxic effects and anticancer potential of the following mycotoxins were described: ergotamine, cyclopiazonic acid, T-2 toxin, satratoxin H, alternariol, pseurotin, synerazol, rubratoxin, beauvericin, enniatin, tenuazonic acid, cytochalasin B, cytochalasin C, and MT81 [32].

The aspects of classifications of the International Agency for Research on Cancer (IARC) (Lyon, France), categorizing human carcinogenic mycotoxins, should be kept in mind (International Agency for Research on Cancer, Lyon, France, 2021) when the cytotoxicity of a Janus-faced mycotoxin is considered as a potential antineoplastic agent. The IARC I category most carcinogenic to humans (e.g., aflatoxins) should be definitively excluded from the list of candidates. The IARC 2A group is probably carcinogenic to humans but provides strong evidence of anticarcinogenicity, making it likely that carcinogenesis is mediated by mechanisms that also operate in humans. These compounds should, therefore, not be kept on the waiting list of antitumor mycotoxins. The IARC 2B group is possibly carcinogenic to humans, although there is limited evidence of carcinogenicity and less than sufficient evidence of anticarcinogenicity. This group deserves consideration but only after the mechanism of carcinogenicity has been ruled out. The IARC 3 group is not classifiable as to its carcinogenicity to humans but is potentially carcinogenic in experimental animals, whereas the mechanism of carcinogenicity does not work in humans. Agents in group 3 and other mycotoxins that do not fall into any other group mentioned are regarded as safe overall, but this does not mean that further research can be avoided.

Due to the inherent toxicity of gentamicins (mainly ototoxicity and nephrotoxicity) that is associated with their long-term use, as well as the emergence of resistant bacterial strains, these molecules can also be regarded as Janus-faced molecules owing to their limited usage. The applications of some aminoglycosides have been extended, as they display clinically relevant activity against protozoa, Neisseria gonorrhoeae, and mycobacteria. 

The recent emergence of infections due to Gram-negative bacteria and bacterial resistance has prompted a revived interest in the use of aminoglycosides while addressing the bacterial resistance, nephrotoxicity, and ototoxicity problems associated with their clinical use [121]. Major fractions of the gentamicin complex (C1, C1a, C2, C2a) possess weak antifungal activity. The cytotoxicity of aminoglycosides against plant pathogenic fungi is indicated by their >25 µg/mL MIC values and they are not recommended for human use. This is the reason aminoglycosides can be regarded as ’Janus-faced’ molecules. Among the minor components (A, A1–A4, B, B1, X), gentamicin B1 showed strong antifungal activity in vitro (0.4–6.2 µg/mL MIC) [1], and moderate but noticeable activity in vivo against plant pathogenic fungi [122]. Consequently, by knowing the structures, the substituents, and the functional groups of aminoglycosides, it should be possible to improve their antifungal activity. The best example is gentamicin B1 itself. Its low in vitro MIC values suggest that gentamicin B1 and the family of aminoglycosides merit further study regarding hemo- and cytotoxicity, not only in inhibitory but also in the killing range. Gentamicin is in medical use and its minor fraction, gentamicin B1, deserves to be tested against systemic plant mycoses that have no efficient medical treatment. Another issue is the interaction between gentamicin B1 and amphotericin B and the exact mechanism of action of this minor component of gentamicins merits study at the chemical level.

## 6. Conclusions

Based on the arrangement of structural elements of ’Janus-faced’ molecules, the following factors have been determined regarding mycotoxins:

**-**  Cytotoxicity is attributable to the presence of multiple hydroxyl groups. In poly-hydroxylated benzenes, toxicity is associated with hydrophobicity and the probability of radical formation [123];
**-**  The IARC carcinogenic property is related to polyene structure (e.g., aflatoxins) [36,55];
**-**  Mutagenicity and genotoxicity could be contributed by the simultaneous presence of keto, hydroxy, and cumulative presence of carboxy groups, although the genotoxicity and mutagenicity of ketamines are doubted [124];
**-**  The general tendency of the toxicity of mycotoxins largely depends on the oxidation state, with -OH groups responsible for cytotoxicity, and keto groups for the apoptotic effect. Oxidative stress is a plausible mechanism for mycotoxin-induced toxicity [125,126].

Furthermore, the following factors have been determined regarding aminoglycosides:

**-**  Ring structures are not aromatic and do not contribute to genotoxicity. Examples are most of the vitamins, nucleic acids, enzymes, coenzymes, hormones, and alkaloids containing N-based heterocycles as scaffolds;
**-**  Polyhydroxy and carbonyl (=CO) groups contribute to the oxidation state but are biodegradable;
**-**  Poly(hydroxy acids) are prepared by the self-condensation polymerization of hydroxy acids. They are biodegradable and have the potential to be chemically recycled [127];
**-**  Gentamicins are less toxic than one would expect from their oxygen-containing substituents.

Finally, despite the known cellular interactions of the functional groups, cytotoxic antifungal agents have not been given much attention. Our results call attention to those cytotoxic ’Janus-faced’ molecules that could be used in tumour treatments despite their moderate genotoxicity. 

## Data Availability

Data is contained within the article.
